# A new *Megophrys* Kuhl & Van Hasselt (Amphibia, Megophryidae) from southeastern China

**DOI:** 10.3897/zookeys.904.47354

**Published:** 2020-01-16

**Authors:** Bin Wang, Yan-Qing Wu, Jun-Wei Peng, Sheng-Chao Shi, Ning-Ning Lu, Jun Wu

**Affiliations:** 1 CAS Key Laboratory of Mountain Ecological Restoration and Bioresource Utilization and Ecological Restoration Biodiversity Conservation Key Laboratory of Sichuan Province, Chengdu Institute of Biology, Chinese Academy of Sciences, Chengdu 610041, China Chengdu Institute of Biology, Chinese Academy of Sciences Chengdu China; 2 Nanjing Institute of Environmental Sciences, Ministry of Ecology and Environment of China, Nanjing 210042, China Ministry of Ecology and Environment of China Nanjing China; 3 Xianju Biodiversity Development Company Limited, Taizhou 317300, China Xianju Biodiversity Development Company Limited Taizhou China

**Keywords:** Taxonomy, new species, molecular phylogenetic analysis, morphology, Zhejiang Province, China

## Abstract

A new species of the genus *Megophrys* from Zhejiang Province, China is described. Molecular phylogenetic analyses supported the new taxon as an independent clade nested into the *Megophrys* clade and sister to *M. lishuiensis*. The new species could be distinguished from its congeners by a combination of the following morphological characteristics: (1) small size (SVL 31.0–36.3 mm in male and 41.6 mm in female); (2) vomerine ridge present and vomerine teeth absent; (3) tongue not notched behind; (4) a small horn-like tubercle at the edge of each upper eyelid; (5) tympanum distinctly visible, rounded; (6) two metacarpal tubercles in hand; (7) relative finger lengths: II < I < IV < III; (8) toes with rudimentary webbing at bases; (9) heels overlapping when thighs are positioned at right angles to the body; (10) tibiotarsal articulation reaching tympanum to eye when leg stretched forward; (11) an internal single subgular vocal sac in male; (12) in breeding male, the nuptial pads with black nuptial spines on the dorsal bases of the first and second fingers.

## Introduction

Megophryidae Bonaparte, 1850 (Amphibia: Anura) is a large Asian toad family which was divided into three subfamilies, i.e., Leptobrachiinae Dubois, 1983, Leptolalaginae Delorme, Dubois, Grosjean & Ohler, 2006 and Megophryinae Bonaparte, 1850 (Delorme et al. 2006). The generic and/or subgeneric classifications in Megophryinae have been under debates for a long time (Tian and Hu 1983; Dubois 1987 “1986”; Lathrop 1997; Rao and Yang 1997; Dubois and Ohler 1998; Jiang et al. 2003; Delorme et al. 2006; Frost et al. 2006; Fei and Ye 2009, 2012, 2016; Chen et al. 2017; Mahony et al. 2017; Liu et al. 2018; Munir et al. 2018) though recent phylogenetic studies clustered all recognised species of Megophryinae into one big clade (e.g., Chen et al. 2017; Mahony et al. 2017; Li et al. 2018). Mahony et al. (2017) classified all members of Megophryinae into a single genus *Megophrys* Kuhl & Van Hasselt, 1822 including seven subgenera (*Megophrys*, *Xenophrys* Günther, 1864, *Panophrys* Rao & Yang, 1997, *Atympanophrys* Tian & Hu, 1983, *Ophryophryne* Boulenger, 1908, *Pelobatrachus* Beddard, 1908 (1907), and *Brachytarsophrys* Tian & Hu, 1983) based on molecular phylogenetics and morphological comparisons. Partly based on this point, Frost (2019) placed all recognised species of Megophryinae into the genus *Megophrys* but with no subgeneric classifications.

The genus *Megophrys* is widely distributed from eastern China through the eastern and southern Himalayas, throughout mainland Indochina and the islands of the Sunda shelf in Indonesia and parts of the Philippines (Frost 2019). It currently contains 92 recognised species (Frost 2019), and noticeably, 37 species were described in the last decade (See species list of *Megophrys* in Frost 2019). The genus is suggested to harbour both cryptic diversity and highly localised species diversification (Chen et al. 2017; Mahony et al. 2017, 2018; Liu et al. 2018). For example, in the genus, just in China, 41 cryptic species were suggested by molecular phylogenetic analyses in Liu et al. (2018), and six of them were subsequently described (Li et al. 2018; Wang et al. 2019). To present, 48 *Megophrys* species have been described in China. Obviously, we still need to verify that undescribed taxa and conduct deep investigations for exploring underestimated diversity in this group especially based on detailed morphological comparisons, molecular phylogenetics and bioacoustics data.

During our field surveys in the Xianju County, Zhejiang Province, China, some *Megophrys* specimens were collected from the montane forests. Molecular phylogenetic analyses and morphological comparisons support that it is distinctly different from its *Megophrys* congeners. Therefore, we describe it herein as a new species.

## Materials and methods

### Specimens

**Figure 1.**
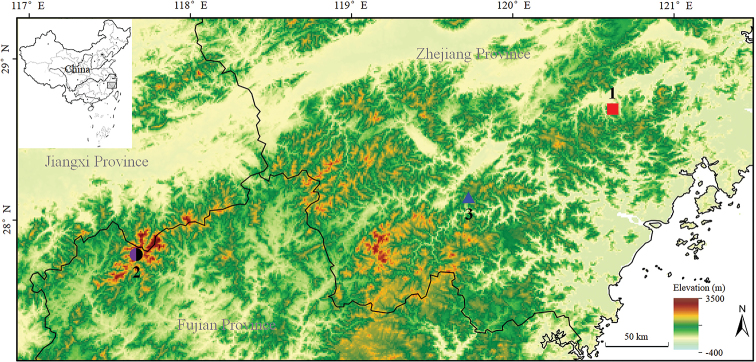
Localities for specimens used in this study. 1, the type locality of *Megophrys xianjuensis* sp. nov., Xianju County, Zhejiang Province, China; 2. the common type locality of *M. boettgeri* (black) and *M. kuatunensis* (purple), Wuyi Mountain, Nanping County, Fujian Province, China; 3, the type locality of *M. lishuiensis*, Lishui City, Zhejiang Province, China.

Seven adult males, two adult females, and six tadpoles of the new taxon (for voucher information see Suppl. material 1: Table S1 and Suppl. material 2: Table S2) were collected from the mountain streams of Xianju County, Zhejiang Province, China (Fig. 1). The stages of tadpoles were identified following Gosner (1960). After taking photographs, they were euthanised using isoflurane, and the specimens were then fixed in 75% ethanol. Tissue samples were taken and preserved separately in 95% ethanol prior to fixation. Specimens were deposited in Chengdu Institute of Biology, Chinese Academy of Sciences (**CIB**, **CAS**).

### Molecular data and phylogenetic analyses

Four male, two female, and three tadpole specimens of the new taxon were included in the molecular analyses (for voucher information see Table 1). For phylogenetic analyses, two topotypes of *M. kuatunensis* and two topotypes of *M. boettgeri* from Wuyi Mountain, Fujian Province, China (the common type locality of these species; Fig. 1) were also collected and sequenced (for voucher information see Table 1).

**Table 1.** Localities, voucher information, and GenBank accession numbers for molecular samples used in this study.

Species		Voucher number	Locality	GenBank accession number	
16S	COI		
*Megophrys aceras*	KIZ025467	Khao Nan National Park, Nakhon Si Thammarat, Thailand	KX811925	KX812159
*M. acuta*	SYS a001957	Heishiding Nature Reserve, Guangdong Prov., China	KJ579118	–
*M. auralensis*	NCSM 79599	Aural, Kampong Speu, Cambodia	KX811807	–
*M. baluensis*	ZMH A13125	Gunung Kinabalu National Park, Kogopan Trail, Malaysia	KJ831310	–
*M. baolongensis*	KIZ019216	Baolong, Chongqing City, China	KX811813	KX812093
*M. binchuanensis*	KIZ019441	Mt. Jizu, Yunnan Prov., China	KX811849	KX812112
*M. binlingensis*	KIZ025807	Mt. Wawu, Sichuan Prov., China	KX811852	KX812115
*M. boettgeri*	Tissue ID: YPXJK033	Mt. Wuyi, Fujian Prov., China	KX811814	KX812104
	CIBWY18082307	Mt. Wuyi, Fujian Prov., China	MN563762	MN563778
	CIBWY18082603	Mt. Wuyi, Fujian Prov., China	MN563763	MN563779
*M. brachykolos*	ROM 16634	Hong Kong, China	KX811897	KX812150
*M. carinense*	Tissue ID: YPX20455	Dayao Shan, Guangxi Prov., China	KX811811	KX812057
*M. cheni*	SYS a001427	Jinggang Shan, Jiangxi Prov., China	KJ560391	–
*M. chuannanensis*	CIB20050081	Hejiang, Sichuan Prov., China	KM504261	–
*M. daweimontis*	KIZ048997	Dawei Shan, Yunnan Prov., China	KX811867	KX812125
*M. dongguanensis*	SYS a001972	Mt. Yinping, Guangdong Prov., China	MK524098	MK524129
*M. dringi*	UNIMAS 8943	Gunung Mulu National Park, Sarawak, Malaysia	KJ831317	–
*M. edwardinae*	FMNH 273694	Bintulu, Sarawak, Malaysia	KX811918	KX812050
*M. elfina*	ZMMU ABV-00454	Bidoup Mountain, Lam Dong, Vietnam	KY425379	–
*M. fansipanensis*	VNMN 2018.01	Lao Cai, Sa Pa, Vietnam	MH514886	–
*M. feae*	KIZ046706	Huangcaoling, Yunnan Prov., China	KX811810	KX812056
*M. flavipunctata*	SDBDU2009.297	East Khasi Hills dist., Meghalaya	KY022307	MH647536
*M. gerti*	ITBCZ 1108	Nui Chua National Park, Ninh Thuan, Vietnam	KX811917	KX812161
*M. gigantica*	SYSa003933	Wuliang shan, Yunnan Prov., China	MH406775	MH406235
*M. glandulosa*	KIZ048439	Husa, Yunnan Prov., China	KX811762	KX812075
*M. hansi*	KIZ010360	Phong Dien Nature Reserve, Thua Thien Hue, Vietnam	KX811913	KX812155
*M. himalayana*	SDBDU2009.75	East Siang dist., Arunachal Pradesh, India	KY022311	–
*M. hoanglienensis*	VNMN 2018.02	Lao Cai, Sa Pa, Vietnam	MH514889	–
*M. huangshanensis*	KIZ022004	Mt. Huang, Anhui Prov., China	KX811821	KX812107
*M. intermedia*	ZFMK 87596	U Bo, Phong Nha-Ke Bang NP, Vietnam	HQ588950	–
*M. jingdongensis*	KIZ-LC0805067	Huanglianshan, Yunnan Prov., China	KX811872	KX812131
*M. jinggangensis*	KIZ07132	Chashan Forest Farm, Jiangxi Prov., China	KX811840	KX812108
*M. jiulianensis*	SYS a002107	Mt. Jiulian, Jiangxi Prov., China	MK524099	MK524130
*M. kalimantanensis*	MZB. Amph 21482	Tanah Bumbu, Kalimantan Selatan, Borneo, Indonesia	MG993554	–
*M. kobayashii*	UNIMAS 8148	Gunung Kinabalu National Park, Sabah, Malaysia	KJ831313	–
*M. kuatunensis*	CIBWY18082407	Mt. Wuyi, Fujian Prov., China	MN563764	MN563780
	CIBWY18082408	Mt. Wuyi, Fujian Prov., China	MN563765	MN563781
	SYS a001579	Mt. Wuyi, Fujian Prov., China	KJ560376	–
*M. lancip*	MZB:Amp:22233	–	KY679891	–
*M. leishanensis*	CIBLS20171101001	Mt. Leigong, Guizhou Prov., China	MK005310	MK005306
*M. ligayae*	ZMMU NAP-05015	Palawan, Philippines	KX811919	KX812051
*M. lini*	SYS a002370	Suichuan Co., Jiangxi Prov., China	KJ560412	–
*M. lishuiensis*	CIBWYF00169	Lishui City, Zhejiang Prov., China	KY021418	–
	CIBWYF00170	Lishui City, Zhejiang Prov., China	KY113084	–
	CIBWYF11011	Lishui City, Zhejiang Prov., China	KY113085	–
*M. major*	SYSa002961	Zhushihe, Yunnan Prov., China	MH406728	MH406180
*M. mangshanensis*	KIZ021786	Nanling National Forest Park, Guangdong Prov., China	KX811790	KX812079
*M. maosonensis*	KIZ016045	Xiaoqiaogou Nature Reserve, Yunnan Prov., China	KX811780	KX812080
*M. medogensis*	KIZ06621	Beibeng, Xizang Prov., China	KX811767	KX812082
*M. microstoma*	KIZ048799	Xiaoqiaogou Nature Reserve, Yunnan Prov., China	KX811914	KX812156
*M. minor*	KIZ01939	Qingcheng Shan, Sichuan Prov., China	KX811896	KX812145
*M. montana*	LSUMZ 81916	Sukabumi, Java, Indonesia	KX811927	KX812163
*M. monticola*	SDBDU 2011.1047 [h]	Darjeeling dist., West Bengal, India	KX894679	–
*M. mufumontana*	SYS a006391	Mt. Mufu, Hunan Prov., China	MK524105	MK524136
*M. nankiangensis*	CIB ZYC517	Nanjiang, Sichuan Prov., China	KX811900	–
*M. nankunensis*	SYS a004498	Mt. Nankun, Guangdong Prov., China	MK524108	MK524139
*M. nanlingensis*	SYS a001959	Nanling Nature Reserve, Guangdong Prov., China	MK524111	MK524142
*M. nasuta*	KIZ019419	Malaysia	KX811921	KX812054
*M. obesa*	SYS a002272	Heishiding Nature Reserve, Guangdong Prov., China	KJ579122	–
*M. ombrophila*	KRM18	Mt. Wuyi, Fujian Prov., China	KX856404	–
*M. omeimontis*	KIZ025765	Mt. E’mei, Sichuan Prov., China	KX811884	KX812136
*M. oreocrypta*	BNHS 6046	West Garo Hills dist., Meghalaya	KY022306	–
*M. pachyproctus*	KIZ010978	Beibeng, Xizang Prov., China	KX811908	KX812153
*M. palpebralespinosa*	KIZ011603	Pu Hu Nature Reserve, Thanh Hoa, Vietnam	KX811888	KX812137
*M. parva*	SYSa003042	Zhushihe, Yunnan Prov., China	MH406737	MH406189
*M. periosa*	BNHS 6061	West Kameng dist., Arunachal Pradesh, India	KY022309	MH647528
*M. popei*	SYS a000589	Naling Nature Reserve, Guangdong Prov., China	KM504251	–
*M. sangzhiensis*	Tissue ID: YPX11006	Badagongshan Nature Reserve, Hunan Prov., China	KX811856	KX812117
*M. shapingensis*	KIZ014512	Liziping Nature Reserve, Sichuan Prov., China	KX811904	KX812060
*M. spinata*	KIZ016100	Mt. Leigong, Guizhou Prov., China	KX811864	KX812119
*M. stejnegeri*	KU 314303	Pasonanca Natural Park, Zamboanga, Philippines	KX811922	KX812052
*M. synoria*	FMNH 262778	O’Reang, Mondolkiri, Cambodia	KY022198	–
*M. tuberogranulata*	Tissue ID: YPX10987	Badagongshan Nature Reserve, Hunan Prov., China	KX811823	KX812095
*M. wawuensis*	KIZ025799	Wawu Shan, Sichuan Prov., China	KX811902	KX812062
*M. wugongensis*	SYS a002610	Wugongshan Scenic Area, Jiangxi Prov., China	MK524114	MK524145
*M. wuliangshanensis*	KIZ046812	Huangcaoling, Yunnan Prov., China	KX811881	KX812129
*M. wushanensis*	KIZ045469	Guangwu Mountain, Sichuan Prov., China	KX811838	KX812094
*M. xianjuensis* sp. nov.	CIBXJ190505	Xianju Co., Zhejiang Prov., China	MN563753	MN563769
	CIBXJ20190801	Xianju Co., Zhejiang Prov., China	MN563754	MN563770
	CIBXJ20190802	Xianju Co., Zhejiang Prov., China	MN563755	MN563771
	CIBXJ20190803	Xianju Co., Zhejiang Prov., China	MN563756	MN563772
	CIB20180514008	Xianju Co., Zhejiang Prov., China	MN563757	MN563773
	CIBXJ190503	Xianju Co., Zhejiang Prov., China	MN563758	MN563774
	CIBXJT19050702	Xianju Co., Zhejiang Prov., China	MN563759	MN563775
	CIBXJT19050703	Xianju Co., Zhejiang Prov., China	MN563760	MN563776
	CIBXJT19050704	Xianju Co., Zhejiang Prov., China	MN563761	MN563777
*M. zhangi*	KIZ014278	Zhangmu, Xizang Prov., China	KX811765	KX812084
*Leptobrachium boringii*	Tissue ID: YPX37539	Emei Shan, Sichuan Prov., China	KX811930	KX812164
*L. oshanensis*	KIZ025778	Emei Shan, Sichuan Prov., China	KX811928	KX812166

Total DNA was extracted using a standard phenol-chloroform extraction protocol (Sambrook et al. 1989). Two fragments of the mitochondrial genes 16S rRNA and cytochrome oxidase subunit I (COI) were amplified. For 16S, the primers P7 (5’-CGCCTGTTTACCAAAAACAT-3’) and P8 (5’-CCGGTCTGAACTCAGATCACGT-3’) were used following Simon et al. (1994), and for COI, Chmf4 (5’-TYTCWACWAAYCAYAAAGAYATCGG-3’) and Chmr4 (5’-ACYTCRGGRTGRCCRAARAATCA-3’) were used following Che et al. (2012). Gene fragments were amplified under the following conditions: an initial denaturing step at 95 °C for 4 min; 36 cycles of denaturing at 95 °C for 30 sec, annealing at 52 °C (for 16S)/47 °C (for COI) for 40 sec and extending at 72 °C for 70 sec. Sequencing was conducted using an ABI3730 automated DNA sequencer in Shanghai DNA BioTechnologies Co., Ltd. (Shanghai, China). New sequences were deposited in GenBank (for GenBank accession numbers see Table 1).

For molecular analyses, the available sequence data for all related species of the genus *Megophrys* were downloaded from GenBank, mainly from previous studies (Chen et al. 2017; Mahony et al. 2017; Liu et al. 2018; Wang et al. 2019; for GenBank accession number see Table 1). For phylogenetic analyses, corresponding sequences of one *Leptobrachella oshanensis* and one *Leptobrachium boringii* were downloaded (for GenBank accession number see Table 1) and used as outgroups according to Chen et al. (2017).

Sequences were assembled and aligned using the Clustalw module in BioEdit v. 7.0.9.0 (Hall 1999) with default settings. Alignments were checked by eye and revised manually if necessary. To avoid bias in alignments, GBLOCKS v. 0.91.b (Castresana 2000) with default settings was used to extract regions of defined sequence conservation from the length-variable 16S gene fragments. Non-sequenced fragments were defined as missing loci.

Phylogenetic trees were reconstructed for the concatenated data of the mitochondrial genes. Phylogenetic analyses were conducted using maximum likelihood (ML) and Bayesian Inference (BI) methods, implemented in PhyML v. 3.0 (Guindon et al. 2010) and MrBayes v. 3.12 (Ronquist and Huelsenbeck 2003), respectively. To avoid under- or over-parameterisation (McGuire et al. 2007), the best partition scheme and the best evolutionary model for each partition were chosen for the phylogenetic analyses using PARTITIONFINDER v. 1.1.1 (Robert et al. 2012). For this analysis, 16S rRNA and COI genes were defined, and Bayesian Inference Criteria (BIC) was used. As a result, the analyses suggested that the best partition scheme was 16S/COI gene, and selected GTR+I+G model as the best model for all partitions. For the ML tree, branch supports were drawn from 10,000 non-parametric bootstrap replicates. In BI analyses, two runs each with four Markov chains were run for 60 million generations with sampling every 1000 generations. The first 25% of generations were removed as the “burn-in” stage followed by calculation of Bayesian posterior probabilities and the 50% majority-rule consensus of the post burn-in trees sampled at stationarity. Finally, genetic distance was calculated with the pairwise uncorrected *p*-distance model between the new taxon and its congeners by 16S rRNA gene using MEGA v. 6.06 (Tamura et al. 2013).

### Morphological comparisons

Nine adult specimens of the new taxon and the holotype and seven paratypes of *M. lishuiensis* were measured (for voucher information see Suppl. material 1: Table S1). The terminology and methods followed Fei et al. (2009). Measurements were taken with a dial calliper to 0.1 mm. In total, 22 morphometric characters of adult specimens were measured:

**ED** eye diameter (distance from the anterior corner to the posterior corner of the eye);

**FIIIL** third finger length (distance from base to tip of finger III);

**FIIL** second finger length (distance from base to tip of finger II);

**FIL** first finger length (distance from base to tip of finger I);

**FIVL** fourth finger length (distance from base to tip of finger IV);

**FL** foot length (distance from tarsus to the tip of fourth toe);

**HAL** hand length (distance from the posterior end of the inner metacarpal tubercle to the distal tip of finger III);

**HDL** head length (distance from the tip of the snout to the articulation of jaw);

**HDW** maximum head width (greatest width between the left and right articulations of jaw);

**IND** internasal distance (minimum distance between the inner margins of the external nares);

**IOD** interorbital distance (minimum distance between the inner edges of the upper eyelids);

**LAL** length of lower arm and hand (distance from the elbow to the distal end of the finger III);

**LW** lower arm width (maximum width of the lower arm);

**SL** snout length (distance from the tip of the snout to the anterior corner of the eye);

**SNT** distance from the tip of the snout to the naris;

**SVL** snout-vent length (distance from the tip of the snout to the posterior edge of the vent);

**TFL** length of foot and tarsus (distance from the tibiotarsal articulation to the distal end of the Toe IV);

**THL** thigh length (distance from vent to knee);

**TL** tibia length (distance from knee to tarsus);

**TW** maximal tibia width;

**TYD** maximal tympanum diameter;

**UEW** upper eyelid width (greatest width of the upper eyelid margins measured perpendicular to the anterior-posterior axis).

For seven tadpoles of the new taxon (for voucher information see Suppl. material 2: Table S2), eleven morphometric characters were measured:

**BH** maximum body height;

**BW** maximum body width;

**IOD** interocular distance (minimum distance between eye);

**MW** mouth width (distance between two corners of mouth);

**SL** snout length (distance from the tip of the snout to the anterior corner of the eye);

**SS** snout to spiraculum (distance from spiraculum to the tip of the snout);

**SVL** snout-vent length;

**TAH** tail height (maximum height between upper and lower edges of tail);

**TAL** tail length (distance from base of vent to the tip of tail);

**TBW** maximum width of tail base;

**TOL** total length (distance from the tip of the snout to the tip of tail).

In order to reduce the impact of allometry, a size-corrected value from the ratio of each character to SVL was calculated and then log-transformed for the following morphometric analyses. One-way analysis of variance (ANOVA) was used to test the significance of differences on morphometric characters between different sexes as well as between different species. The significance level was set at 0.05. Furthermore, to show the spatial distribution of each species on the morphometric characters, principal component analyses (PCA) were performed. These analyses were carried out in R (R Development Core Team 2008).

We compared morphological characters of the new taxon with other *Megophrys* species. Comparative data were obtained from the literature for 92 species of the genus (Table 2). In addition, for comparison, we examined the type and/or topotype materials for *M. lishuiensis*, *M. boettgeri*, and *M. kuatunensis* (for voucher numbers see Table 1 and Suppl. material 1: Table S1).

**Table 2.** References for morphological characters for congeners of the genus *Megophrys*.

Species	Literature obtained
*M. aceras* Boulenger, 1903	Taylor 1962
*M. acuta* Wang, Li & Jin, 2014	Li et al. 2014
*M. ancrae* Mahony, Teeling & Biju, 2013	Mahony et al. 2013
*M. auralensis* Ohler, Swan & Daltry, 2002	Ohler et al. 2002
*M. baluensis* (Boulenger, 1899)	Boulenger 1899
*M. baolongensis* Ye, Fei & Xie, 2007	Ye et al. 2007
*M. binchuanensis* Ye & Fei, 1995	Ye and Fei 1995
*M. binlingensis* Jiang, Fei & Ye, 2009	Fei et al. 2009
*M. boettgeri* (Boulenger, 1899)	Fei et al. 2012
*M. brachykolos* Inger & Romer, 1961	Inger and Romer 1961
*M. carinense* (Boulenger, 1889)	Fei et al. 2009
*M. caudoprocta* Shen, 1994	Fei et al. 2012
*M. cheni* (Wang & Liu, 2014)	Wang et al. 2014
*M. chuannanensis* (Fei, Ye & Huang, 2001)	Fei et al. 2012
*M. damrei* Mahony, 2011	Mahony 2011
*M. daweimontis* Rao & Yang, 1997	Fei et al. 2012
*M. dongguanensis* Wang & Wang, 2019	Wang et al. 2019
*M. dringi* Inger, Stuebing & Tan, 1995	Inger et al. 1995
*M. edwardinae* Inger, 1989	Inger 1989
*M. elfina* Poyarkov, Duong, Orlov, Gogoleva, Vassilieva, Nguyen, Nguyen, Nguyen, Che & Mahony, 2017	Poyarkov et al. 2017
*M. fansipanensis* Tapley, Cutajar, Mahony, Nguyen, Dau, Luong, Le, Nguyen, Nguyen, Portway, Luong & Rowley, 2018	Tapley et al. 2018
*M. feae* Boulenger, 1887	Fei et al. 2009
*M. feii* Yang, Wang & Wang, 2018	Yang et al. 2018
*M. flavipunctata* Mahony, Kamei, Teeling & Biju, 2018	Mahony et al. 2018
*M. gerti* (Ohler, 2003)	Ohler 2003
*M. gigantica* Liu, Hu & Yang, 1960	Fei et al. 2012
*M. glandulosa* Fei, Ye & Huang, 1990	Fei et al. 2012
*M. hansi* (Ohler, 2003)	Ohler 2003
*M. himalayana* Mahony, Kamei, Teeling & Biju, 2018	Mahony et al. 2018
*M. hoanglienensis* Tapley, Cutajar, Mahony, Nguyen, Dau, Luong, Le, Nguyen, Nguyen, Portway, Luong & Rowley, 2018	Tapley et al. 2018
*M. huangshanensis* Fei & Ye, 2005	Fei et al. 2012
*M. insularis* (Wang, Liu, Lyu, Zeng & Wang, 2017)	Wang et al. 2017a
*M. intermedia* Smith, 1921	Rao and Yang 1997
*M. jingdongensis* Fei & Ye, 1983	Fei et al. 2012
*M. jinggangensis* (Wang, 2012)	Wang et al. 2012
*M. jiulianensis* Wang, Zeng, Lyu & Wang, 2019	Wang et al. 2019
*M. kalimantanensis* Munir, Hamidy, Matsui, Iskandar, Sidik & Shimada, 2019	Munir et al. 2019
*M. kobayashii* Malkmus & Matsui, 1997	Malkmus and Matsui 1997
*M. koui* Mahony, Foley, Biju & Teeling, 2017	Mahony et al. 2017
*M. kuatunensis* Pope, 1929	Fei et al. 2012
*M. lancip* Munir, Hamidy, Farajallah & Smith, 2018	Munir et al. 2018
*M. leishanensis* Li, Xu, Liu, Jiang, Wei & Wang, 2018	Li et al. 2018
*M. lekaguli* Stuart, Chuaynkern, Chan-ard & Inger, 2006	Stuart et al. 2006
*M. liboensis* (Zhang, Li, Xiao, Li, Pan, Wang, Zhang & Zhou, 2017)	Zhang et al. 2017
*M. ligayae* Taylor, 1920	Taylor 1920
*M. lini* (Wang & Yang, 2014)	Wang et al. 2014
*M. lishuiensis* (Wang, Liu & Jiang, 2017)	Wang et al. 2017b
*M. longipes* Boulenger, 1886	Taylor 1962
*M. major* Boulenger, 1908	Mahony et al. 2018
*M. mangshanensis* Fei & Ye, 1990	Fei et al. 2012
*M. maosonensis* Bourret, 1937	Bourret 1937
*M. medogensis* Fei, Ye & Huang, 1983	Fei et al. 2012
*M. megacephala* Mahony, Sengupta, Kamei & Biju, 2011	Mahony et al. 2011
*M. microstoma* (Boulenger, 1903)	Fei et al. 2012
*M. minor* Stejneger, 1926	Fei et al. 2012
*M. montana* Kuhl & Van Hasselt, 1822	Kuhl & Van Hasselt 1822
*M. monticola* (Günther, 1864)	Mahony et al. 2018
*M. mufumontana* Wang, Lyu & Wang, 2019	Wang et al. 2019
*M. nankiangensis* Liu & Hu, 1966	Fei et al. 2012
*M. nankunensis* Wang, Zeng &. Wang, 2019	Wang et al. 2019
*M. nanlingensis* Lyu, Wang, Liu & Wang, 2019	Wang et al. 2019
*M. nasuta* (Schlegel, 1858)	Taylor 1962
*M. obesa* Wang, Li & Zhao, 2014	Wang et al. 2014
*M. ombrophila* Messenger & Dahn, 2019	Munir et al. 2019
*M. omeimontis* Liu, 1950	Fei et al. 2009
*M. oreocrypta* Mahony, Kamei, Teeling & Biju, 2018	Mahony et al. 2018
*M. oropedion* Mahony, Teeling & Biju, 2013	Mahony et al. 2013
*M. pachyproctus* Huang, 1981	Fei et al. 2009
*M. palpebralespinosa* Bourret, 1937	Fei et al. 2012
*M. parallela* Inger & Iskandar, 2005	Inger and Iskandar 2005
*M. parva* (Boulenger, 1893)	Fei et al. 2009
*M. periosa* Mahony, Kamei, Teeling & Biju, 2018	Mahony et al. 2018
*M. popei* (Zhao, Yang, Chen, Chen & Wang, 2014)	Zhao et al. 2014
*M. robusta* Boulenger, 1908	Mahony et al. 2018
*M. rubrimera* Tapley, Cutajar, Mahony, Chung, Dau, Nguyen, Luong & Rowley, 2017	Tapley et al. 2017
*M. sangzhiensis* Jiang, Ye & Fei, 2008	Jiang et al. 2008
*M. serchhipii* (Mathew & Sen, 2007)	Mathew and Sen 2007
*M. shapingensis* Liu, 1950	Fei et al. 2009
*M. shuichengensis* Tian & Sun, 1995	Fei et al. 2009
*M. shunhuangensis* Wang, Deng, Liu, Wu & Liu, 2019	Wang et al. 2019
*M. spinata* Liu & Hu, 1973	Fei et al. 2009
*M. stejnegeri* Taylor, 1920	Taylor 1920
*M. synoria* (Stuart, Sok & Neang, 2006)	Stuart et al. 2006
*M. takensis* Mahony, 2011	Mahony 2011
*M. tuberogranulata* Shen, Mo & Li, 2010	Fei et al. 2012
*M. vegrandis* Mahony, Teeling, Biju, 2013	Mahony et al. 2013
*M. wawuensis* Fei, Jiang & Zheng, 2001	Fei et al. 2012
*M. wugongensis* Wang, Lyu & Wang, 2019	Wang et al. 2019
*M. wuliangshanensis* Ye & Fei, 1995	Fei et al. 2012
*M. wushanensis* Ye & Fei, 1995	Fei et al. 2012
*M. zhangi* Ye & Fei, 1992	Fei et al. 2012
*M. zunhebotoensis* (Mathew & Sen, 2007)	Mathew and Sen 2007

### Bioacoustics notes

The advertisement calls of the new taxon from Xianju County, Zhejiang Province, China were recorded in the field. SONY PCM-D50 digital sound recorder was used to record within 20 cm of the calling individuals. The sound files in wave format were resampled at 48 kHz with sampling depth 24 bits. The sonograms and waveforms were generated by WaveSurfer software (Sjöander and Beskow 2000) from which all parameters and characters were measured. Ambient temperature was taken by a digital hygrothermograph.

## Results

### Phylogenetic analyses

**Figure 2.**
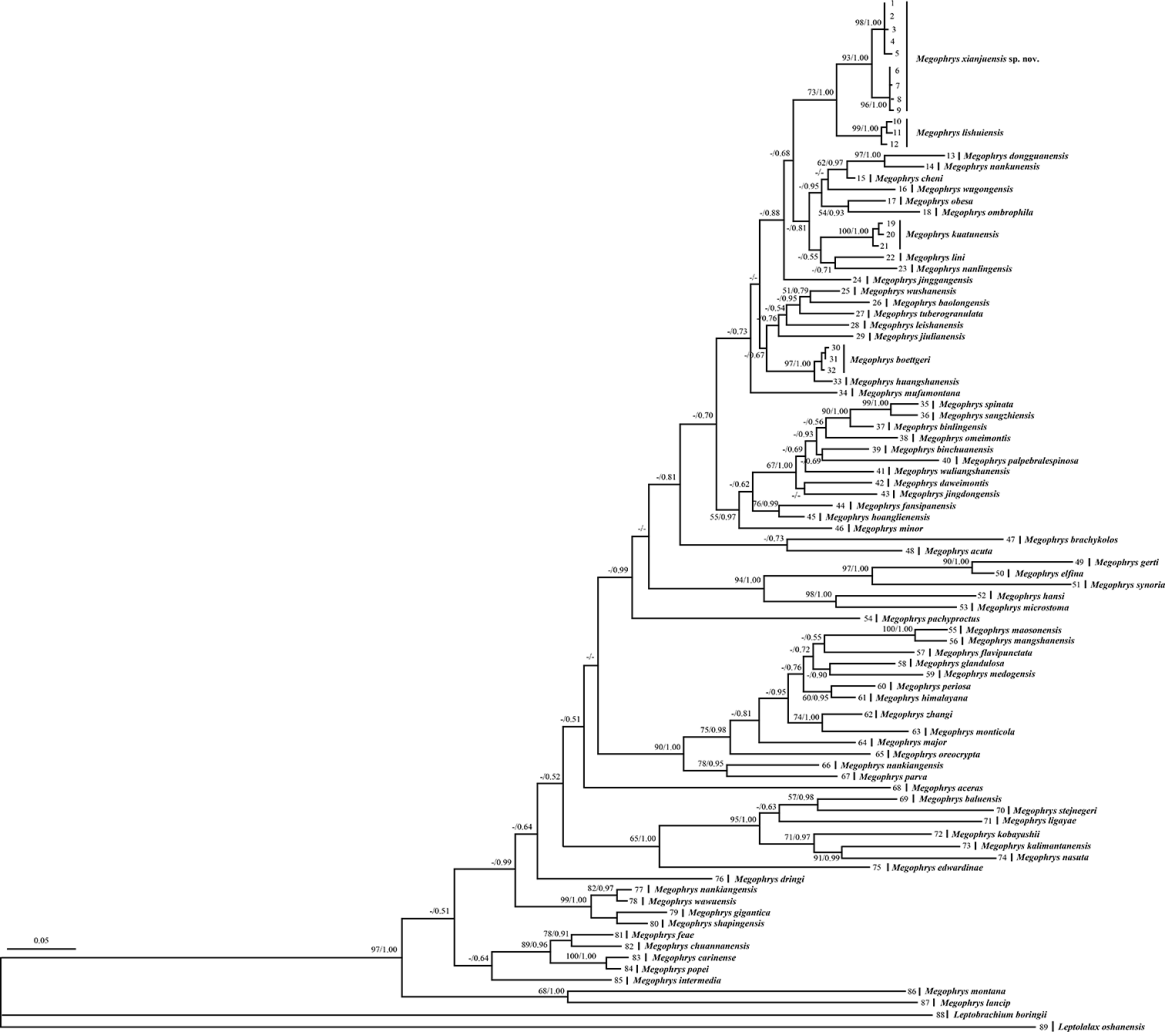
Maximum Likelihood tree of the genus *Megophrys* reconstructed based on the 16S rRNA and COI gene sequences. ML bootstrap support/Bayesian posterior probability was denoted beside each node. Samples 1-89 refer to Table 1.

Aligned sequence matrix of 16S+COI contained 1104 bps. ML and BI trees of the mitochondrial DNA dataset presented almost consistent topology, though relationships of some lineages were unresolved (Fig. 2). All individuals of the new taxon were clustered into one clade which was independently nested into the *Megophrys* clade and sister to the *M. lishuiensis* clade. Genetic distances on 16S gene with uncorrected *p*-distance model between specimens of the new taxon were less than 0.7% (ranging from 0.0% to 0.6%), being lower than most interspecific genetic distances in the genus *Megophrys* (0.4%–19.3%; Suppl. material 3: Table S3). Genetic distance between the new taxon and its closely-related species *M. lishuiensis* is 2.8%, higher than that between many substantial species, for example, *M. wushanensis* vs. *M. baolongensis* (2.1%), *M. baolongensis* vs. *M. tuberogranulata* (1.5%), *M. sangzhiensis* vs. *M. binlingensis* (1.7%), and *M. omeimontis* vs. *M. jingdongensis* (2.1%).

### Morphometric analyses

**Figure 3.**
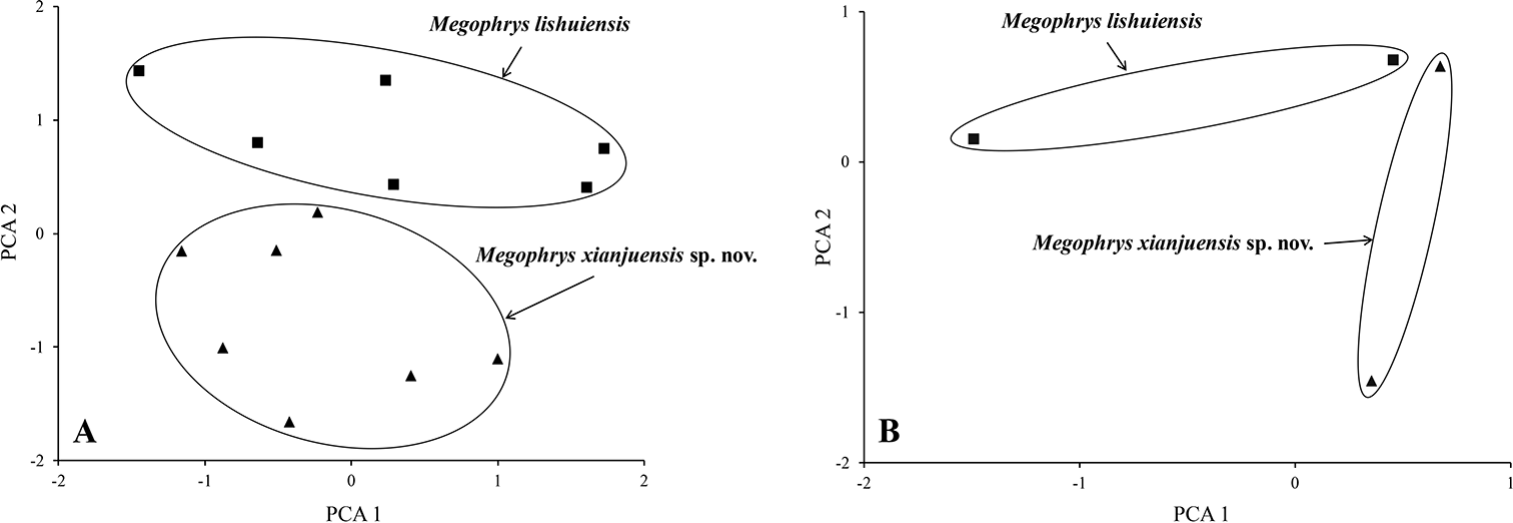
Plots of the first principal component (PC1) versus the second (PC2) for *Megophrys xianjuensis* sp. nov. and *M. lishuiensis* from principal component analyses **A** male **B** female.

The results of one-way ANOVA showed that in the new taxon, the male group was significantly different from the female group on SVL and the ratio of ED and IND to SVL (*p*-value < 0.05). Therefore, morphometric analyses between the new taxon and *M. lishuiensis* were conducted separately for male and female. In PCA, the total variation of the first two principal components was 56.2% in male and 74.9% in female respectively. In both male and female, the new taxon could be distinctly separated from its phylogenetically sister species *M. lishuiensis* on the two-dimensional plots of PC1 vs. PC2 (Fig. 3). The results of one-way ANOVA indicated that the new taxon was significantly different from *M. lishuiensis* on many morphometric characters (all *p*-values < 0.05; Table 3). More detailed descriptions of results from morphological comparisons between the new taxon and its congeners were presented in the following sections for describing the new species.

**Table 3.** Morphometric comparisons between the adult specimens of *Megophrys xianjuensis* sp. nov. and *M. lishuiensis*. Units are in mm. See abbreviations for the morphological characters in Materials and methods section.

**Character**	***Megophrys xianjuensis* sp. nov. (MX)**				***M. lishuiensis* (ML)**				***P*-value from ANOVA**		
	**Males (*N* = 7)**		**Female (*N* = 2)**		**Males (*N* = 6)**		**Female (*N* = 2)**				
	**Range**	**Mean ± SD**	**Range**	**Mean ± SD**	**Range**	**Mean ± SD**	**Range**	**Mean ± SD**	**Male vs. female in MX**	**MX vs. ML in male**	**MX vs. ML in female**
SVL	31.0–36.3	33.1 ± 1.8	32.8–41.6	37.2 ± 6.2	30.5–34.7	32.5 ± 1.6	37.6–40.4	39.0 ± 1.9	0.119	0.633	0.728
HDL	9.4–10.5	9.9 ± 0.4	10.1–10.8	10.4 ± 0.5	9.4–10.3	9.8 ± 0.4	9.9–11.4	10.6 ± 1.1	0.365	0.916	0.705
HDW	10.6–11.5	11.1 ± 0.3	10.7–12.9	11.8 ± 1.5	10.6–11.6	10.9 ± 0.3	12.1–12.1	12.1 ± 0.02	0.361	0.813	0.561
SL	3.8–4.7	4.2 ± 0.3	3.6–5.2	4.4 ± 1.1	4.1–4.5	4.3 ± 0.2	4.3–4.4	4.3 ± 0.1	0.318	0.569	0.510
SNT	1.9–2.5	2.2 ± 0.2	1.8–2.5	2.1 ± 0.5	2.0–2.5	2.3 ± 0.2	2.4–2.7	2.6 ± 0.2	0.165	0.177	0.177
IND	3.2–3.7	3.4 ± 0.2	3.2–3.6	3.4 ± 0.3	3.1–4.0	3.5 ± 0.4	4.2–4.2	4.2 ± 0.01	0.045*	0.470	0.121
IOD	2.3–3.3	2.8 ± 0.4	2.8–2.9	2.8 ± 0.1	2.4–2.8	2.5 ± 0.1	2.8–3.1	2.9 ± 0.2	0.345	0.140	0.959
UEW	2.3–2.9	2.7 ± 0.2	2.7–3.4	3.1 ± 0.5	2.6–3.6	3.2 ± 0.3	3.7–3.7	3.7 ± 0.02	0.807	0.004**	0.047*
ED	3.3–4.0	3.6 ± 0.3	2.9–4.0	3.4 ± 0.8	3.8–4.6	4.1 ± 0.2	4.1–4.6	4.3 ± 0.3	0.002**	0.002**	0.046*
TYD	1.8–2.2	2.0 ± 0.1	1.7–2.7	2.2 ± 0.6	1.9–2.5	2.1 ± 0.2	2.2–2.3	2.3 ± 0.1	0.571	0.044*	0.962
LAL	14.2–17.2	15.3 ± 1.0	13.7–19.2	16.5 ± 3.9	14.6–15.7	15.2 ± 0.4	17.5–17.6	17.5 ± 0.04	0.279	0.792	0.767
HAL	7.6–9.2	8.2 ± 0.6	7.8–10.0	8.9 ± 1.5	8.0–8.8	8.4 ± 0.3	8.7–9.3	9.0 ± 0.4	0.489	0.220	0.021*
LW	2.1–2.7	2.5 ± 0.2	2.0–2.6	2.3 ± 0.4	2.4–3.1	2.8 ± 0.2	2.4–2.4	2.4 ± 0.01	0.062	0.042*	0.879
FIL	2.5–3.1	2.9 ± 0.2	2.8–3.9	3.4 ± 0.78	2.4–3.0	2.7 ± 0.2	2.8–3.6	3.2 ± 0.5	0.488	0.644	0.602
FIIL	2.7–3.5	3.0 ± 0.3	3.0–4.0	3.5 ± 0.78	2.5–3.3	2.9 ± 0.3	3.0–3.2	3.1 ± 0.2	0.675	0.975	0.151
FIIIL	4.4–6.2	4.9 ± 0.6	4.8–5.9	5.4 ± 0.8	4.9–5.7	5.2 ± 0.2	5.3–5.8	5.6 ± 0.3	0.622	0.111	0.949
FIVL	2.9–4.1	3.3 ± 0.2	3.0–4.4	3.6 ± 1.0	3.2–3.9	3.5 ± 0.2	3.2–3.6	3.4 ± 0.3	0.726	0.110	0.471
THL	13.8–16.0	14. ± 0.8	13.1–17.6	15.3 ± 3.2	13.7–14.7	14.2 ± 0.4	15.7–16.3	16. 0 ± 0.4	0.177	0.646	0.996
TL	14.5–16.1	15.0 ± 0.5	13.0–17.8	15.4 ± 3.4	14.3–15.5	15.0 ± 0.3	16.4–16.7	16.5 ± 0.2	0.019*	0.558	0.628
TW	2.8–4.4	3.4 ± 0.6	2.9–3.3	3.1 ± 0.3	3.9–4.4	4.1 ± 0.2	4.0–4.7	4.3 ± 0.5	0.124	0.004**	0.050
TFL	18.0–21.5	19.5 ± 1.14	19.3–23.5	21.4 ± 3.0	18.6–20.4	19.7 ± 0.7	21.0–22.4	21.7 ± 1.0	0.483	0.444	0.697
FL	11.2–14.8	12.9 ± 1.1	12.4–15.6	14.0 ± 2.2	12.3–13.5	12.840 ± 0.5	13.7–14.8	14.3 ± 0.8	0.562	0.946	0.734

### Taxonomic accounts

#### Megophrys xianjuensis sp. nov.

Taxon classificationAnimaliaAnuraMegophryidae

017CE79C-8EED-5983-A18D-843C0D96A304

http://zoobank.org/8CFD24E0-1ABD-4642-B9C1-FFA1D80262C2

Figures 1 , 2 , 3 , 4 , 5 , 6 , 7 , 8 , 9

##### Type material.

**Figure 4.**
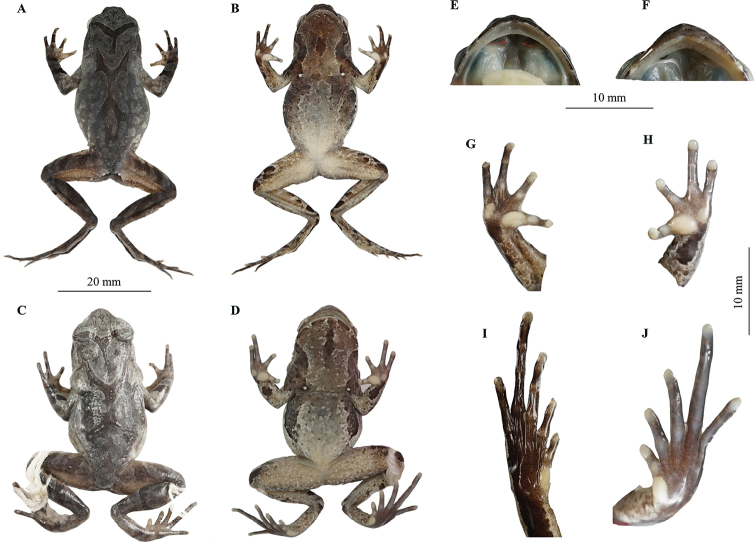
Holotype CIBXJ190503 of *Megophrys xianjuensis* sp. nov. and holotype CIBWYF00164 of *M. lishuiensis***A, B** dorsal view and ventral of *Megophrys xianjuensis* sp. nov., respectively **C, D** dorsal view and ventral of *M. lishuiensis*, respectively **E** oral of *Megophrys xianjuensis* sp. nov. showing vomerine ridges (red arrows) **F** oral of *M. lishuiensis* showing no vomerine ridge **G** ventral view of hand of *Megophrys xianjuensis* sp. nov. **H** ventral view of hand of *M. lishuiensis***I** ventral view of foot of *Megophrys xianjuensis* sp. nov. **J** ventral view of foot of *M. lishuiensis*.

***Holotype*.** CIBXJ190503 (Fig. 4A, B, E, G, I), adult male, from Shenxianju scenic area, Danzhu Township, Xianju County, Zhejiang Province, China (28.677483N, 120.594888E, 350 m a. s. l.), collected by Bin Wang on 7 May 2019.

***Paratype*.** One adult male CIB20180514007 and one adult female CIB20180514008 collected from the type locality of the new species by Bin Wang on 14 May 2018; one adult male CIBXJ190501 and one adult female CIBXJ190505 collected from the type locality by Bin Wang on 7 May 2019; five adult males CIBXJ20190801, CIBXJ20190802, CIBXJ20190803, CIBXJ20190804 and CIBXJ20190805 collected from the type locality by Bin Wang on 28 August 2019.

##### Diagnosis.

*Megophrys xianjuensis* sp. nov. is assigned to the genus *Megophrys* based on molecular phylogenetic analyses and the following generic diagnostic characters: snout shield-like; projecting beyond the lower jaw; canthus rostralis distinct; chest gland small and round, closer to the axilla than to midventral line; femoral gland on rear of thigh; vertical pupils.

The new species could be identified from its congeners by a combination of the following morphological characters: (1) small size (SVL 31.0–36.3 mm in males and 41.6 mm in female); (2) vomerine ridge present and vomerine teeth absent; (3) tongue not notched behind; (4) a small horn-like tubercle at the edge of each upper eyelid; (5) tympanum distinctly visible, rounded; (6) two metacarpal tubercles in hand; (7) relative finger lengths: II < I < IV < III; (8) toes with rudimentary webbing at bases; (9) heels overlapping when thighs are positioned at right angles to the body; (10) tibiotarsal articulation reaching tympanum to eye when leg stretched forward; (11) an internal single subgular vocal sac in male; (12) in breeding male, the nuptial pads with black nuptial spines on the dorsal bases of the first and second fingers.

##### Description of holotype.

SVL 34.4 mm; head wider than long (HDW/HDL ratio 1.2); snout obtusely pointed, protruding well beyond the margin of the lower jaw in ventral view; loreal region vertical and concave; canthus rostralis well developed; top of head flat on in dorsal view; an small horn-like tubercle at the edge of the upper eyelid; eye large and convex, eye diameter 40.4% of head length; pupils vertical; nostril orientated laterally, closer to snout than eye; tympanum distinct, TYP/EYE ratio 0.56; vomerine ridges present and vomerine teeth absent; margin of tongue smooth, not notched behind (Fig. 4A, B, E).

Forelimbs slender, the length of lower arm and hand 44.9% of SVL; fingers slender, relative finger lengths: II < I < IV < III; tips of digits globular, without lateral fringes; subarticular tubercle distinct at the base of each fingers; two metacarpal tubercles, prominent, oval-shaped, the inner one bigger than the outer (Fig. 4G).

Hindlimbs slender, heels overlapping when thighs are positioned at right angles to the body, tibiotarsal articulation reaching tympanum to eye when leg stretched forward; tibia length slightly longer than thigh length; relative toe lengths I < II < V < III < IV; tips of toes round, slightly dilated; subarticular tubercle absent; toes with rudimentary webbing at bases; lateral fringe narrow; inner metatarsal tubercle oval-shaped; outer metatarsal tubercle absent (Fig. 4I).

Dorsal rough, with numerous granules; several large warts scattered on flanks; an small horn-like tubercle at the edge of each upper eyelid; a dark brown inverted triangular pattern between anterior corner of eyes, tubercles on the dorsum forming a weak X-shaped ridge and two discontinuous dorsolateral parallel ridges on either side of the X-shaped ridge; several tubercles on the flanks and dorsal surface of thighs and tibias, and limbs barred with dark brown forming four transverse rows; supratympanic fold distinct (Fig. 4A).

Ventral surface with numerous white granules; chest gland distinct and round, closer to the axilla than to midventral line; femoral gland on rear of thigh; posterior end of the body protrudes distinct and appears as an arc-shaped swelling, upper the anal region (Fig. 4B).

##### Colouration of holotype in life.

**Figure 5.**
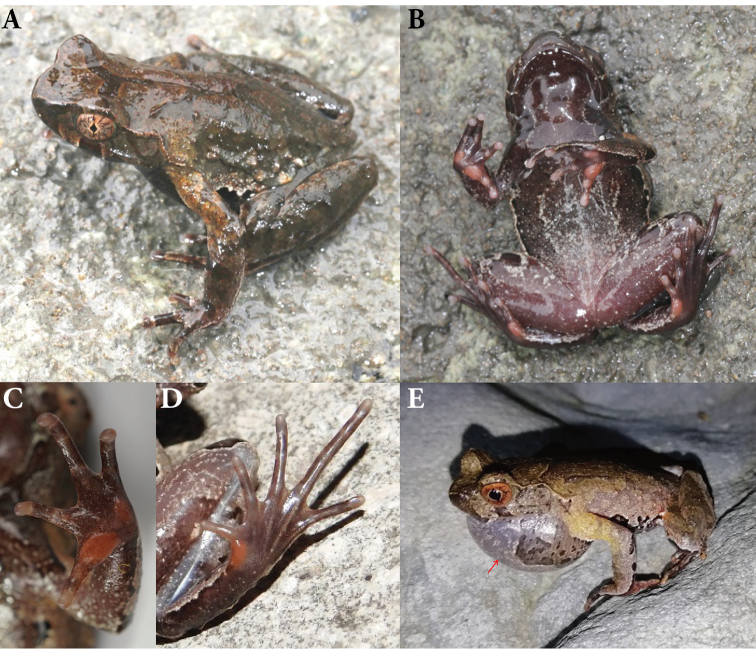
Photos of the holotype CIBXJ190503 of *Megophrys xianjuensis* sp. nov. in life **A** dorsal view **B** ventral view **C** ventral view of hand **D** ventral view of foot **E** dorsolateral view showing the single external subgular vocal sac (red arrow).

An inverted triangular brown speckle between the eyes; an X-shaped ridges on the dorsum of body, four transverse bands on the dorsal surface of the hindlimb; several dark brown and white vertical bars on the lower and upper lip; the venter purple and the colour of throat is deeper than belly, flank and middle of throat with black brown spots, numerous white granules on the ventral surface and limbs; palms and soles purple and the metacarpal tubercles are orange, tip of digits greyish white; pectoral and femoral glands white (Fig. 5).

##### Preserved holotype colouration.

Dorsal surface fade to olive; the inverted triangular brown speckle between the eyes, X-shaped ridges on dorsum and transverse bands on limbs and digits and brown spots on flank and middle throat are more distinct; ventral surface greyish white; creamy-white substitutes the orange in metacarpal tubercles; the posterior of ventral surface of body, inner of thigh and upper of tibia creamy-white (Fig. 4).

##### Variation.

**Figure 6.**
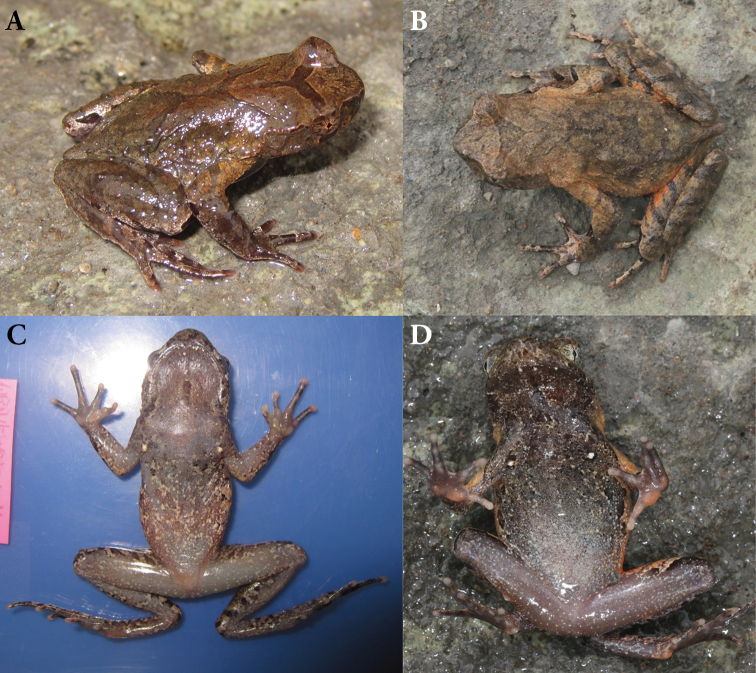
Colour variation in *Megophrys xianjuensis* sp. nov. in life **A, B** dorsolateral view of a male (Voucher: CIBXJ190501) and a female (Voucher: CIBXJ190505), respectively **C, D** ventral view of CIBXJ190501 and CIBXJ190505, respectively.

In some adult individuals a brown Y-shaped marking on the dorsum of head and disconnected with a X-shaped marking on back (Fig. 6A); an inverted triangular brown speckle between two upper eyelids with Y-shaped marking on back of trunk, the colouration of dorsum is brown with brick-red and the caudal vertebra is pointed in the adult female (Fig. 6B); in some adult individuals the metacarpal tubercles in palm is grey-white but the tip of fingers is orange and the black spots in flank is smaller (Fig. 6C); in some adult individuals the white granules in ventral surface are more intensive (Fig. 6D).

##### Advertisement calls.

**Figure 7.**
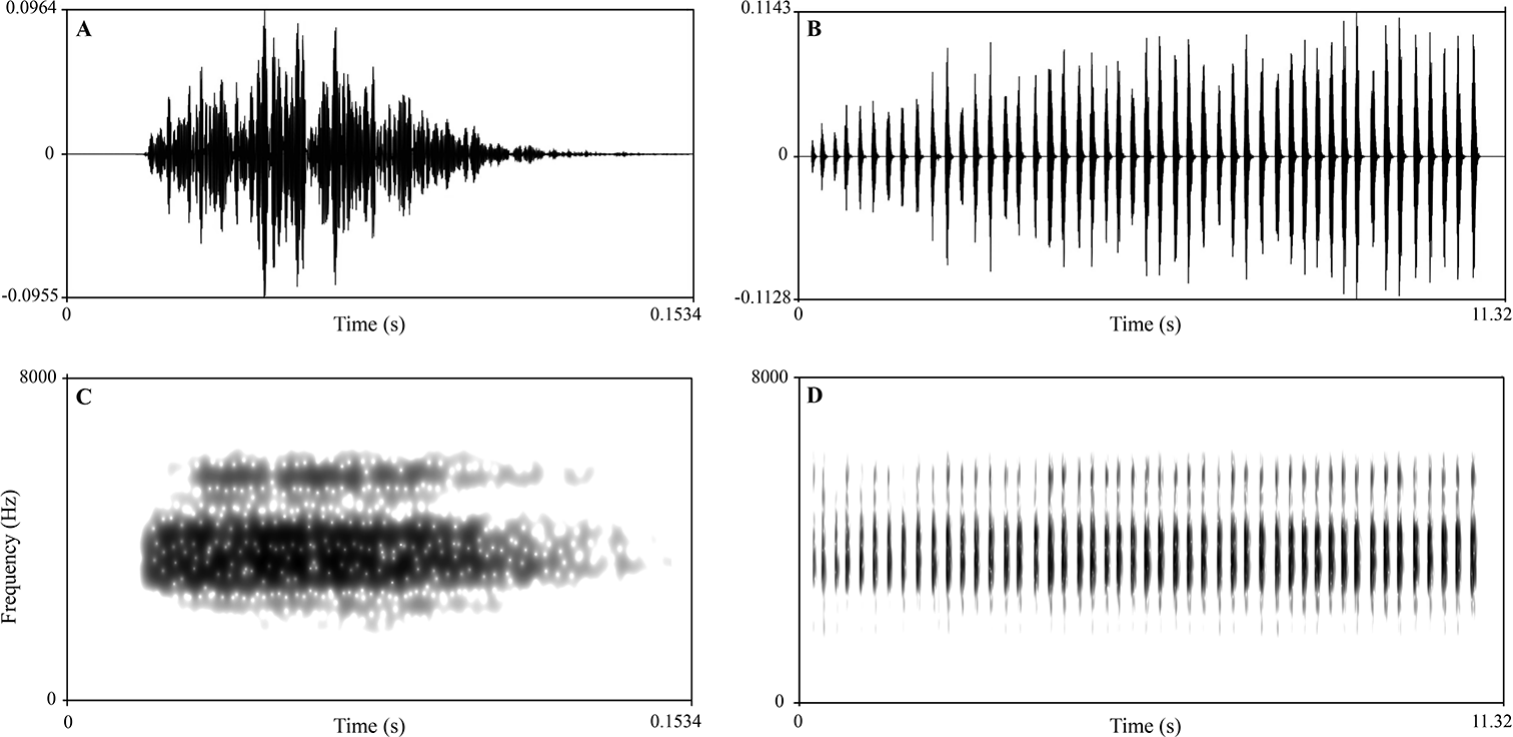
Advertisement call of the holotype CIBXJ190503 of *Megophrys xianjuensis* sp. nov. **A** waveform showing one note **B** waveform showing three notes of one advertisement call **C** sonogram showing one note **D** sonogram showing three notes of one call.

Ten advertisement calls from two individuals of the new species were recorded in the Xianju County, Zhejiang Province, China between 21:00–23:00 on 7 May 2019. The call description is based on recordings of the holotype CIBXJ090503 (Fig. 7) from the stone near the streamlet, and the ambient air temperature was 24.5 °C. Each call consists of 22–62 (mean 49.33 ± 14.50, *N* = 6) notes. Call duration was 4.90–14.22 second (mean10.95 ± 3.42, *N* = 6). Call interval was 4.12–7.64 second (mean 6.23 ± 1.56, *N* = 5). Each note had a duration of 0.06– 0.90 second (mean 0.10 ± 0.05, *N* = 290) and the intervals between notes 0.07–0.88 second (mean 0.13 ± 0.05, *N* = 284). Amplitude modulation within note was apparent, beginning with lower energy pulses, increasing slightly to a maximum by approximately mid note, and then decreasing towards the end of each note. The average dominant frequency was 6400 ± 465.79 (5520–6840 Hz, *N* = 6).

##### Secondary sexual characteristics.

Adult females with SVL 41.6 mm, larger than adult males with 31.0–36.3 mm. Adult males have a single subgular vocal sac (Fig. 5E). In breeding males, the brownish red nuptial pads on the dorsal bases of the first finger and second fingers with black nuptial spines under microscope.

##### Tadpole description.

**Figure 8.**
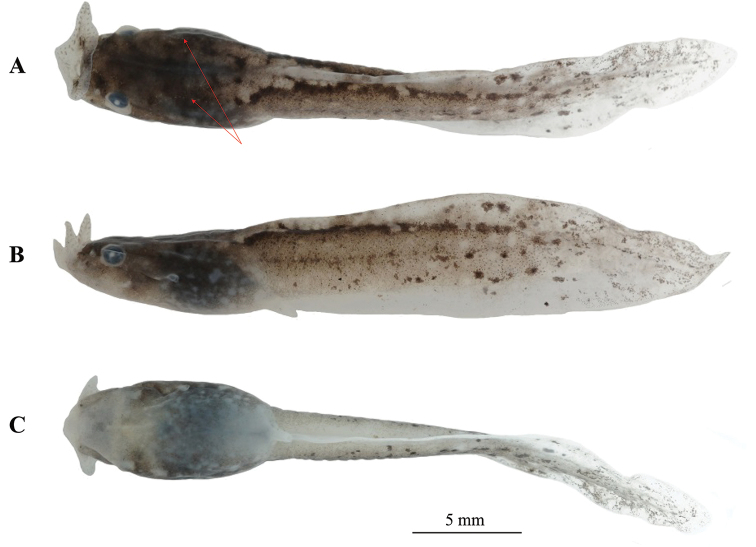
Tadpole specimen CIBXJT19050704 of *Megophrys xianjuensis* sp. nov. **A** dorsal view **B** lateral view **C** ventral view. Arrows indicate two dotted lines on flank of dorsum from mouth to fin.

The tadpole was confirmed as *Megophrys xianjuensis* sp. nov. by molecular phylogenetic analyses. The following tadpole description is based on the specimen CIBXJT19050704 at Stage 31 (Fig. 8). Body slender, body and tail yellow-brown in life and fade to light brown and creamy-white of body and tail respectively in preserved specimens; two dotted lines on flank of dorsal from mouth to fin; tail height greater than body height; dorsal fin arising, behind the origin of the tail, height near mid-length, tapering gradually to narrow, tip pointed; tail approximately 1.9 times as long as snout-vent length; tail height 19.9% of tail length; body width slightly longer than body height (BW/BH = 1.1); tail fins lightly coloured, tail muscles with small black spots; eyes large, lateral, nostril near eyes; spiracle on the left side of the body and distinct; oral disk terminal, lips expanded and directed upwardly into a umbelliform oral disk; transverse width of expanded funnel 18.1% of snout-vent length.

##### Morphological comparisons.

By having small size body, *Megophrys xianjuensis* sp. nov. differs from *M. aceras*, *M. auralensis*, *M. binlingensis*, *M. carinense*, *M. caudoprocta*, *M. chuannanensis*, *M. damrei*, *M. edwardinae* (in female), *M. feae*, *M. flavipunctata*, *M. gigantica*, *M. glandulosa*, *M. himalayana*, *M. intermedia*, *M. jingdongensis*, *M. kobayashii*, *M. kalimantanensis*, *M. lekaguli*, *M. liboensis*, *M. ligayae*, *M. longipes*, *M. major*, *M. mangshanensis*, *M. maosonensis*, *M. medogensis*, *M. omeimontis*, *M. oreocrypta*, *M. periosa*, *M. popei*, *M. robusta*, *M. spinata*, *M. sangzhiensis*, *M. shapingensis* and *M. shuichengensis* and *M. takensis* (maximum SVL < 42.0 mm in the new species vs. minimum SVL > 45 mm in the latter).

By lacking vomerine teeth, *Megophrys xianjuensis* sp. nov. differs from *M. aceras*, *M. ancrae*, *M. carinense*, *M. baluensis*, *M. caudoprocta*, *M. chuannanensis*, *M. damrei*, *M. daweimontis*, *M. dongguanensis*, *M. fansipanensis*, *M. flavipunctata*, *M. glandulosa*, *M. hoanglienensis*, *M. himalayana*, *M. insularis*, *M. intermedia*, *M. jingdongensis*, *M. jinggangensis*, *M. jiulianensis*, *M. kalimantanensis*, *M. kobayashii*, *M. lancip*, *M. lekaguli*, *M. liboensis*, *M. ligayae*, *M. major*, *M. mangshanensis*, *M. maosonensis*, *M. medogensis*, *M. megacephala*, *M. montana*, *M. nasuta*, *M. nankunensis*, *M. nanlingensis*, *M. omeimontis*, *M. oropedion*, *M. oreocrypta*, *M. palpebralespinosa*, *M. parallela*, *M. parva*, *M. periosa*, *M. popei*, *M. robusta*, *M. rubrimera*, *M. sangzhiensis*, *M. stejnegeri*, *M. takensis*, *M. zhangi* and *M. zunhebotoensis* (vs. present in the latter).

By having a small horn-like tubercle at the edge of each upper eyelid, *Megophrys xianjuensis* sp. nov. differs from *M. binchuanensis*, *M. binlingensis*, *M. damrei*, *M. gigantica*, *M. minor*, *M. nasuta*, *M. nankiangensis*, *M. oropedion*, *M. pachyproctus*, *M. spinata*, *M. stejnegeri*, *M. takensis*, *M. wuliangshanensis*, *M. wushanensis*, *M. zhangi*, and *M. zunhebotoensis* (vs. tubercle lacking in the latter) and differs from *M. carinense*, *M. feae*, *M. gerti*, *M. hansi*, *M. intermedia*, *M. kalimantanensis*, *M. koui*, *M. liboensis*, *M. microstoma*, *M. palpebralespinosa*, *M. popei*, *M. shuichengensis*, and *M. synoria* (vs. having a prominent and elongated tubercle at the edge of each upper eyelid in the latter).

With its tongue not notched behind, *Megophrys xianjuensis* sp. nov. differs from *M. ancrae*, *M. baolongensis*, *M. binlingensis*, *M. boettgeri*, *M. carinense*, *M. cheni*, *M. chuannanensis*, *M. damrei*, *M. dringi*, *M. fansipanensis*, *M. feae*, *M. feii*, *M. flavipunctata*, *M. gerti*, *M. glandulosa*, *M. hoanglienensis*, *M. huangshanensis*, *M. insularis*, *M. jiulianensis*, *M. jingdongensis*, *M. kalimantanensis*, *M. kuatunensis*, *M. liboensis*, *M. mangshanensis*, *M. maosonensis*, *M. medogensis*, *M. minor*, *M. nankiangensis*, *M. nanlingensis*, *M. omeimontis*, *M. oropedion*, *M. pachyproctus*, *M. parallela*, *M. popei*, *M. robusta*, *M. sangzhiensis*, *M. shapingensis*, *M. shuichengensis*, *M. spinata*, *M. vegrandis*, *M. wawuensis*, *M. zhangi*, and *M. zunhebotoensis* (vs. tongue notched behind in the latter).

By toes rudimentary webbing at base, *Megophrys xianjuensis* sp. nov. differs from *M. brachykolos*, *M. carinense*, *M. flavipunctata*, *M. jingdongensis*, *M. jinggangensis*, *M. lini*, *M. major*, *M. palpebralespinosa*, *M. popei*, *M. shuichengensis*, and *M. spinata* (vs. at least one-fourth webbed in the latter).

By heels overlapping when thighs are positioned at right angles to the body, *Megophrys xianjuensis* sp. nov. differs from *M. acuta*, *M. brachykolos*, *M. dongguanensis*, *M. huangshanensis*, *M. kuatunensis*, *M. nankunensis*, *M. obesa*, *M. ombrophila*, and *M. wugongensis* (vs. heels not meeting when thighs are positioned at right angles to the body in the latter).

By tibiotarsal articulation reaching forward to the region between tympanum and eye when hindlimb is stretched along the side of the body, *Megophrys xianjuensis* sp. nov. differs from *M. baolongensis*, *M. nankiangensis*, *M. pachyproctus*, *M. serchhipii*, *M. shuichengensis*, and *M. tuberogranulata* (vs. reaching posterior corner of the eye in the latter); differs from *M. daweimontis*, *M. glandulosa*, *M. lini*, *M. major*, *M. medogensis*, *M. obesa*, and *M. sangzhiensis* (vs. reaching the anterior corner of the eye or beyond eye or nostril and tip of snout in the latter); differs from *M. leishanensis* (vs. reaching middle part of eye); differs from *M. mufumontana* (vs. reaching tympanum in males and to the eye in females).

By having an internal single subgular vocal sac in male, *Megophrys xianjuensis* sp. nov. differs from *M. caudoprocta*, *M. shapingensis*, and *M. shuichengensis* (vs. vocal sac absent in the latter).

By having nuptial pads and nuptial spines on the dorsal base of the first and second fingers in breeding male, *Megophrys xianjuensis* sp. nov. differs from *M. acuta*, *M. feii*, *M. shapingensis*, and *M. shuichengensis* (vs. nuptial pads and nuptial spines lacking in the latter); differs from *M. boettgeri*, and *M. elfina* (vs. nuptial pads and nuptial spines only on the first finger in the latter).

*Megophrys boettgeri* and *M. kuatunensis* were suggested to be distributed in Zhejiang Province, China and might be sympatric with *Megophrys xianjuensis* sp. nov. (Fei et al. 2016; Wang et al. 2017b). The new species can be distinguished from these species by a series of morphological characters as follows. The new species vs. *M. boettgeri*: vomerine ridges present vs. vomerine ridges absent, tibiotarsal articulation reaching forward to the region between tympanum and eye when hindlimb is stretched along the side of the body vs. tibiotarsal articulation reaching forward to eye, having nuptial pads and nuptial spines on the dorsal base of the first and second fingers in breeding male vs. nuptial pads and nuptial spines only on the first finger, heels overlapping when thighs are positioned at right angles to the body vs. heels just meeting, light round patches on the shoulder absent vs. present. The new species vs. *M. kuatunensis*: heels overlapping when thighs are positioned at right angles to the body vs. heels not meeting, toes with rudimentary webbing at bases vs. toes without webbing.

Molecular phylogenetic analyses revealed that the new species was genetically closer to *M. lishuiensis*. The new species can be distinguished from *M. lishuiensis* by a series of morphological characters as follows. Vomerine ridges present vs. vomerine ridges absent; heels overlapping when thighs are positioned at right angles to the body vs. heels just meeting or not meeting; light round patches on the shoulder absent vs. present; having significantly lower ratios of UEW, ED, TYD, LW and TW to SVL in males; and having significantly higher ratios of UEW, ED and HAL to SVL in females (all *p*-values < 0.05; Table 3).

##### Distribution and habitats.

**Figure 9.**
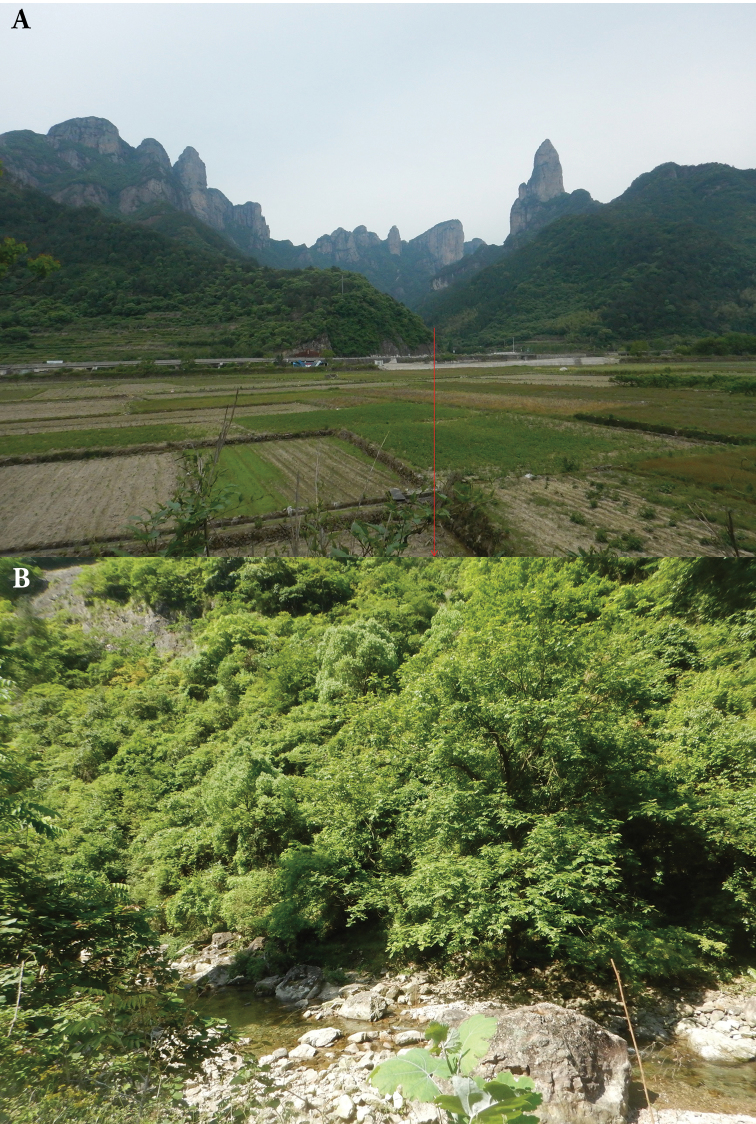
Habitats of *Megophrys xianjuensis* sp. nov. in the type locality, Xianju County, Zhejiang Province, China **A** landscape showing mountain forests **B** a mountain stream where toads of the new species occur.

*Megophrys xianjuensis* sp. nov. is known from the type locality, Xianju County, Zhejiang Province, China at elevations between 320–480 m a.s.l. This new species is frequently found on stones in the streams in the subtropical montane forests (Fig. 5E; Fig. 9). Six sympatric amphibian species, i.e., *Amolops wuyiensis*, *Odorrana tianmuensis*, *O. graminea*, *O. tormota*, *Limnonectes fujianensis*, and *Quasipaa spinosa*, were found.

##### Etymology.

The specific epithet *xianjuensis* refers to Xianju County, Zhejiang Province, China, where the type locality of the species is located. We propose the common name “Xianju horned toad” in English and Xian ju Jiao Chan in Chinese.

## Discussion

Although *Megophrys xianjuensis* sp. nov. superficially resembles *M. lishuiensis*, our integrative comparisons with morphological and molecular data can clearly identify the new species from the latter. This indicates that conserved morphology could hamper species delineation, requiring the incorporation of detailed morphological, genetic, and bioacoustic data to recognise cryptic species. The discovery of *Megophrys xianjuensis* sp. nov. brings the total number of species in the genus to 93, with 49 of them recorded in China (Fei et al. 2016; Frost 2019). Yet, there remain still dozens of undescribed species just in China (Chen et al. 2017; Liu et al. 2018). As noted, the new species was a newly-found clade which has not been reported in previous phylogenetic works although it belongs to the subgenus Panophrys according to our phylogenetic frameworks and previous classifications (Mahony et al. 2017; Liu et al. 2018).

According to records in Fei et al. (2016) and Wang et al. (2017b), *M. boettgeri* is widely distributed in Zhejiang Province, China. However, with two-years’ field surveys and detailed comparisons with many *Megophrys* specimens from Xianju County, Zhejiang Province, we have not found *M. boettgeri* in this region. Similarly, in this county, we have also not found *M. kuatunensis* and *M. lishuiensis* even though they were reported to be distributed in western and southern parts of Zhejiang Province, China (Fei et al. 2016; Wang et al. 2017b); therefore, we suggest that probably in Xianju County, Zhejiang Province, China, there is no *M. boettgeri*, *M. kuatunensis* or *M. lishuiensis*. Considering the high underestimated species level and localised diversification in *Megophrys* (Chen et al. 2017; Liu et al. 2018; Wang et al. 2019), wider and in-depth surveys should be conducted in the eastern part of Zhejiang Province and adjacent areas for detecting distributional range of the new species and for finding cryptic species.

Many *Megophrys* species have narrow distributions fitting the “micro-endemism” model of Liu et al. (2018) and Wang et al. (2019). Similarly, according to our surveys, *Megophrys xianjuensis* sp. nov. is probably only distributed in a narrow range in Xianju County and/or adjacent regions. Hence, this species is likely to be threatened as scenic sites in Xianju County harbouring its habitats (Fig. 9) are increasingly impacted by tourists and considerable developments in tourist infrastructure easily leading to habitat loss. Additionally, an increased frequency of extreme weather events in recent years also influenced the species; for example, the super typhoon “Lekima” in August 2019 causing heavy rain seriously broke many channels and forests where the species occurs in Xianju County. It is urgent to conduct more surveys to understand the population status of the new species and to develop appropriate protection strategies.

## Supplementary Material

XML Treatment for Megophrys xianjuensis

## Supplementary materials

Supplementary material 1 Table S1. Measurements of the adult specimens of *Megophrys xianjuensis* sp. nov. and *M. lishuiensis* Data type: measurements https://binary.pensoft.net/file/372358This dataset is made available under the Open Database License (http://opendatacommons.org/licenses/odbl/1.0/). The Open Database License (ODbL) is a license agreement intended to allow users to freely share, modify, and use this Dataset while maintaining this same freedom for others, provided that the original source and author(s) are credited.Bin Wang, Yan-Qing Wu, Jun-Wei Peng, Sheng-Chao Shi, Ning-Ning Lu, Jun Wu

Supplementary material 2 Table S2. Measurements of the tadpole specimens of *Megophrys xianjuensis* sp. nov. Data type: measurements https://binary.pensoft.net/file/372359This dataset is made available under the Open Database License (http://opendatacommons.org/licenses/odbl/1.0/). The Open Database License (ODbL) is a license agreement intended to allow users to freely share, modify, and use this Dataset while maintaining this same freedom for others, provided that the original source and author(s) are credited.Bin Wang, Yan-Qing Wu, Jun-Wei Peng, Sheng-Chao Shi, Ning-Ning Lu, Jun Wu

Supplementary material 3 Table S3. Uncorrected *p*-distance between *Megophrys* species based on the 16S gene sequences Data type: molecular data https://binary.pensoft.net/file/372360This dataset is made available under the Open Database License (http://opendatacommons.org/licenses/odbl/1.0/). The Open Database License (ODbL) is a license agreement intended to allow users to freely share, modify, and use this Dataset while maintaining this same freedom for others, provided that the original source and author(s) are credited.Bin Wang, Yan-Qing Wu, Jun-Wei Peng, Sheng-Chao Shi, Ning-Ning Lu, Jun Wu
