# Supplementary material for: A new Megophrys Kuhl & Van Hasselt (Amphibia, Megophryidae) from southeastern China
Source: Zookeys. 2020 Jan 16;904:35–62. doi: 10.3897/zookeys.904.47354 (PMC6978424; doi:10.3897/zookeys.904.47354)
Supplement: Supplementary material 2 [file zookeys-904-035-s002.docx]

**Table S2.** Measurements of the tadpole specimens of *Megophrys xianjuensis* sp. nov. Units in mm. See abbreviations for the morphological characters in Materials and methods section.

| **Species** | **Voucher number** | **Stage** | **TOL** | **SVL** | **BW** | **BH** | **SL** | **SS** | **IOD** | **TAL** | **TAH** | **TBW** | **MW** |
| --- | --- | --- | --- | --- | --- | --- | --- | --- | --- | --- | --- | --- | --- |
| *Megophrys xianjuensis* sp. nov. | CIBXJT19050702 | 38 | 35.7 | 13.7 | 5.8 | 4.8 | 3.4 | 7.2 | 3.2 | 22.3 | 6.4 | 3.0 | 2.7 |
| *Megophrys xianjuensis* sp. nov. | CIBXJT19050703 | 38 | 34.9 | 12.6 | 4.1 | 3.3 | 3.0 | 5.7 | 2.2 | 22.5 | 5.1 | 2.6 | 2.0 |
| *Megophrys xianjuensis* sp. nov. | CIBXJT19050704 | 31 | 28.9 | 10.1 | 3.4 | 3.0 | 2.8 | 5.6 | 2.3 | 18.8 | 3.7 | 1.9 | 1.8 |
| *Megophrys xianjuensis* sp. nov. | CIBXJT19050705 | 37 | 35.2 | 12.7 | 5.3 | 4.1 | 3.4 | 7.4 | 3.0 | 22.6 | 5.2 | 2.6 | 2.4 |
| *Megophrys xianjuensis* sp. nov. | CIBXJT19050706 | 37 | 32.7 | 11.7 | 4.5 | 4.2 | 2.8 | 5.3 | 2.7 | 20.7 | 4.9 | 2.4 | 2.2 |
| *Megophrys xianjuensis* sp. nov. | CIBXJT19050707 | 36 | 35.2 | 11.4 | 4.6 | 4.0 | 3.2 | 5.7 | 3.3 | 23.7 | 5.1 | 2.8 | 2.1 |
